# Antisaccadic Eye Movements Are Correlated with Corpus Callosum White Matter Mean Diffusivity, Stroop Performance, and Symptom Burden in Mild Traumatic Brain Injury and Concussion

**DOI:** 10.3389/fneur.2015.00271

**Published:** 2016-01-18

**Authors:** Windsor Kwan-Chun Ting, Tom A. Schweizer, Jane Topolovec-Vranic, Michael D. Cusimano

**Affiliations:** ^1^Injury Prevention Research Office, Keenan Research Centre for Biomedical Science, Li Ka Shing Knowledge Institute, St. Michael’s Hospital, Toronto, ON, Canada; ^2^Neuroscience Research Program, St. Michael’s Hospital, Toronto, ON, Canada; ^3^Department of Surgery, Division of Neurosurgery, St. Michael’s Hospital, Toronto, ON, Canada; ^4^Department of Occupational Science and Occupational Therapy, University of Toronto, Toronto, ON, Canada

**Keywords:** antisaccade, concussion, mild traumatic brain injury, diffusion tensor imaging, executive function, corpus callosum, symptom burden

## Abstract

Antisaccades are thought to involve higher level inputs from neural centers involved in rapid eye movement inhibition and control. Previous work has demonstrated that performance on the antisaccade task can help in the assessment of injury in acute and/or chronic mild traumatic brain injury (mTBI). In this exploratory study, we performed cross-sectional and longitudinal comparisons of rapid eye movement, followed by correlations of antisaccade performance with assessments of symptom burden, diffusion tensor imaging, and a neuropsychological test of response inhibition. Significant deficits in antisaccade median latency, *F*(2, 31) = 3.65, *p* = 0.04 and prosaccade error mean duration, *F*(2, 31) = 3.63, *p* = 0.04 were found between patient groups and controls: the former was correlated with loss of white matter integrity in the splenium of the corpus callosum in acute mTBI, rho = 0.90, *p* = 0.0005. Furthermore, increased antisaccade median latency was also correlated with poor performance on an executive functioning task, *r*^2^ = 0.439, *p* = 0.03, and greater symptom burden, *r*^2^ = 0.480, *p* = 0.02 in the acute mTBI patients. Our preliminary research suggests that the antisaccade task could be useful as a neurological marker for mTBI and concussion, but more work is required.

## Introduction

Few objective assessment tools exist to aid the clinician in the assessment of mild traumatic brain injury (mTBI) and concussion from both an emergency standpoint and in clinic follow-up. Furthermore, little is known about the relationship of symptom load to measures like white matter integrity and other objective measures of brain function. Comprehensive scales have been developed that systematically seek to identify the collective cognitive, emotional, and somatic symptoms after mTBI of all etiologies, but an objective test would be an advantage for assessment. Executive functioning deficits after TBI encompass domains such as attention (1), working memory (2, 3), response inhibition, and other cognitive subsystems involved in the control and regulation of behavior, due to frontal lobe susceptibility to injury. The Stroop color and word test is a common measure of a specific type of executive functioning known as interference control.

For mTBI patients, traumatic axonal injury is thought to be a result of damage to the neurofilament organization and axolemma (4). Diffusion tensor imaging (DTI) findings after mTBI (4) have found that the frontal association white matter tracts, namely, the anterior corpus callosum, superior longitudinal fasciculus, anterior corona radiata, and uncinate fasciculus were common areas of white matter damage in mTBI. Significant effect sizes of FA reductions and MD increases have been found across studies in a meta-analysis of the corpus callosum (5). The corpus callosum, in particular, is important for many cognitive functions; it is involved in bilateral information transfer and coordination between the prefrontal cortices (6). Croall et al. conducted a longitudinal study in mild/moderate TBI patients who were scanned acutely and returned for a chronic assessment, correlating structural integrity with neuropsychological performance, and found significant changes in fractional anisotropy and mean diffusivity in acute mTBI patients (on average 6 days post-mTBI) compared to controls in the ascending and posterior corpus callosum, respectively (7). However, this study did not investigate inhibitory control processes. Kinnunen et al. found significantly lower FA in the mTBI compared to control groups in the corpus callosum and several other areas (8). MD was elevated in the mTBI groups compared to the control groups in similar areas, including the internal and external capsule. The authors of this study did not find significant correlations between executive functioning and FA, but there was a correlation between higher mean diffusivity in the white matter of the superior temporal areas and worse performance on executive functioning tasks. To our knowledge, there does not seem to be any studies directly correlating a subset of executive functioning, namely interference control performance to FA/MD changes in both acute mTBI and persistent post-traumatic symptom (PTS) patients at the same time.

It is important to understand the relationships of symptom burden and executive functioning to antisaccade performance in acute mTBI and PTS, but antisaccades’ relationship to DTI has not been investigated. Specifically, we do not know whether the major white matter pathways damaged in mTBI (corpus callosum, superior longitudinal fasciculus, uncinate fasciculus, and anterior corona radiata) are necessarily common to the white matter pathways necessary for antisaccade performance (projections from the dorsolateral prefrontal cortex, anterior cingulate cortex to the superior colliculus) in mTBI or are related to their function in some way. One focus of this study will be identifying correlations with significant differences in saccade performance variables. There is no literature investigating whether there is any correlation between acute structural integrity (measured by DTI) and performance on the antisaccade.

**Figure 1:**
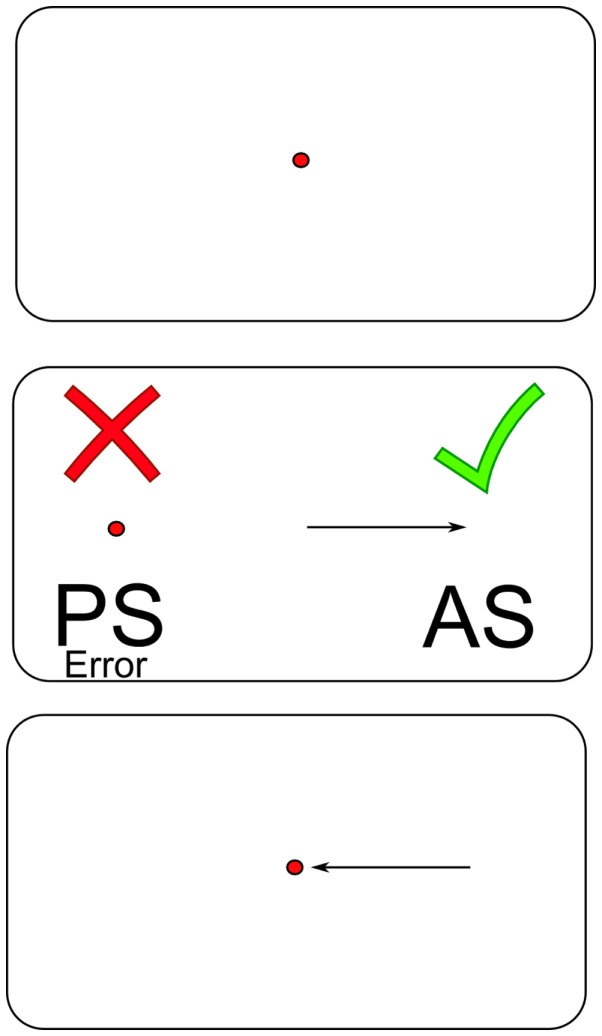
**An illustrated schematic of the antisaccade task**. Abbreviations: PS = prosaccade; AS = antisaccade.

Recent work has advocated the use of rapid eye movements in the assessment of TBI (9). Generating a saccadic eye movement requires coordinated action between many control centers, and the neurophysiology involved has been well defined after decades of study (10–12). Completing the antisaccade task requires making a rapid eye movement to the mirror-opposite location to where a saccade target appears, relative to a central fixation point as soon as possible (Figure 1). The cognitive demands of the color-word component of the Stroop color and word test are similar to those of the antisaccade. Participants must give a verbal response indicating the color ink in which the word is printed, but not what the word says. The word stimulus interferes with the correct verbal response, in much the same way that the prosaccade error target interferes with the correct antisaccade response. Performance on these two tasks should be correlated to some extent. Successful completion of interference tasks requires well-functioning response inhibition control systems from the higher cognitive systems in the frontal and parietal areas of the brain, which may be common to both neuropsychological and antisaccade performance. For the antisaccade task specifically (13), it is thought that the higher cortical regions in the frontal (14) and parietal lobes, namely the frontal eye fields (10, 15), supplementary eye fields, dorsolateral prefrontal cortex (10, 15, 16), posterior parietal cortex, and parietal eye field (10) are responsible for inhibiting the reflexive prosaccade error (17).

Research has suggested that mTBI patients exhibit deficits in saccadic generation and difficulty inhibiting a planned saccadic eye movement within 2 days of injury (18). Antisaccade latency returns to normal after 1 week post-mTBI (19, 20), but accuracy differences have been documented up to few months postinjury (20). Some studies have shown that antisaccade accuracy appears to be impaired in the acute stages, with patients making more errors than controls (19–21); others indicate better performance (22). Only one study was found to relate acute antisaccade performance to neuroimaging, and the focus was mainly on functional performance (21). Functional hyperactivation was observed in the cerebellum and V5/V1 cortical areas, but not the frontal areas during the antisaccade task, contrary to their initial predictions. Antisaccade performance in TBI patients with chronic symptoms several years after injury has been investigated (23). Kraus et al. found significant increases in the number of prosaccade errors (saccade toward stimulus) and latency (in the overlap condition) in the antisaccade task in TBI patients compared with healthy controls but no differences between TBI patients of different severities (mTBI versus moderate/severe TBI). This suggested that there was low specificity of the antisaccade task in differentiating between mTBI and more severe head injuries in the chronic stages, but their use of electrooculography limits the precision of antisaccade measurement compared to the chip-based measurement methods.

Studies to date in PTS patients have not correlated antisaccade performance with gold standard structural neuroimaging. A useful antisaccade marker should be furthermore related to the symptoms that patients are experiencing. Heitger et al. correlated saccade deficiencies in the acute phase with quality of life in the PTS phase (24). Importantly, oculomotor performance (including antisaccade tasks as well as other saccade test results) 1 week after injury was significantly predictive of greater symptom burden on the Rivermead Post-Concussive Symptoms Questionnaire (RPQ) and certain domain scores of the Short-Form-36 questionnaire at 3 and 6 months following mTBI. This study expanded upon Heitger et al.’s earlier results but performed correlations within the same visit, similar to their later work (25) but with a greater number of antisaccade trials per participant.

Previous work has correlated antisaccade performance with a cluster of executive functioning tests and found a significant negative correlation between antisaccade latency and a pooled executive functioning score for a sample of TBI subjects of all severities – only considering the mTBI patients, they found trends (23). Also important is their use of pooled scores for their correlational analyses, which may have masked direct correlations between specific executive functioning tests and antisaccade performance.

Symptom assessment, neuroimaging, and neuropsychological assessment are helpful but insufficient for diagnosing mTBI. Rapid eye movements can be measured after injury, either alone or as an adjunct to the measures previously discussed. Although we have reviewed several eye movements that may be useful for more severe disorders of consciousness inflicted by moderate to severe TBI (26), more information is needed to determine whether antisaccades are related to common assessment metrics in mTBI, and this is a gap in the current literature.

Our primary aim is to confirm whether components of the antisaccade performance are affected by acute mTBI and those with PTS when compared to controls, hypothesizing that antisaccade performance should be most affected early after acute mTBI, it should improve over time, and it should be worse in PTS individuals. Our secondary aim is to examine neuroimaging of these patients, hypothesizing there would be lower fractional anisotropy and increased mean diffusivity in acute and PTS patients’ scans compared to control patients’ scans in white matter regions of interest (ROIs) due to diffuse axonal injury after mTBI. Specific areas of interest included the corpus callosum and all its component areas (genu, body, and splenium), uncinate fasciculus, superior longitudinal fasciculus, internal capsule (part of the anterior corona radiata). Importantly, we hypothesized that antisaccade performance would be correlated with FA and MD in these white matter areas. In our final aim, we hypothesized that patients with poor antisaccade performance would also show poor performance in neuropsychological tests of response inhibition since antisaccades involve inhibitory processes mediated by the frontal and prefrontal cortices. Further, we hypothesized that measures of symptom burden improve after mTBI, and the former would be correlated to antisaccade performance.

## Materials and Methods

All participants provided written informed consent before being prospectively enrolled in the study. We screened patients who registered in the Emergency Department and Outpatient Head Injury Clinic at St. Michael’s Hospital (SMH). Healthy controls were recruited from word of mouth to the friends and relatives of the participants. This work was part of a larger study, which was approved by the Research Ethics Board at SMH.

### Inclusion and Exclusion Criteria

We used the World Health Organization definition of mTBI (27) (see Supplementary Methods in Supplementary Material) with some qualifications. PTS patients sustained mTBI at least 3 months prior to testing and reported PTS 3 months or longer after their injury. We included concussions within our group of mTBI patients. We chose to focus on mTBI and not purely concussion, as positive neuroimaging should not preclude antisaccade measurement if this tool were to be eventually used in the clinic environment. Inclusion criteria for the study also included age greater than or equal to 16 years, able to provide informed consent and adequate verbal English language skills. All participants required a structured history to rule out horizontal diplopia, severe loss of visual acuity and exclusion conditions noted below. The control group was matched to the patient groups based on age (±5 years), sex, and years of education (±5 years).

Acute mTBI, PTS, and healthy control participants were excluded if they were medically unstable or intoxicated (by drugs or alcohol) at the time of recruitment, had medical comorbidities of multiple sclerosis, prior incisional brain surgery, prior brain irradiation, prior hydrocephalus, a history of stroke, early/alcohol-related dementia, neurodegenerative disease including Alzheimer’s disease, and uncontrolled diabetes. In terms of optic pathology, participants were excluded if they had a history of eye disease (amblyopia or glaucoma) or orbital trauma due to the present injury. Participants were excluded from the saccadometry analysis if they were taking medications known to interfere with saccadic eye movement function (benzodiazepines and antipsychotics).

### Participant Assessments

Acute mTBI patients were asked to come in for two visits to SMH: the first visit within a week of injury, and the second visit 2–4 weeks after the injury. Patients with PTS recruited from the head injury clinic were invited to SMH once to participate in all study activities. Healthy control participants who had no history of TBI were recruited and invited to come into SMH twice, 2–4 weeks apart. We attempted to minimize the latency between injury and the first visit. During the first visit, the participant completed an antisaccade task for 100 trials, a series of neuropsychological tests and the MRI scan if scheduling allowed. Prior to this, we obtained demographics information and injury characteristics through taking a brief history. A second assessment was carried out between 2 and 4 weeks after the participant’s mTBI. During the second assessment, we carried out the same tests as we did in the first assessment to assess recovery of function after mTBI on both neuropsychological testing and antisaccade performance. PTS participants conducted all neurological and neuropsychological tests, as well as the MRI on one visit.

### Rapid Eye Movement Measurement

We used a portable research device, the *Saccadometer Advanced* from Ober Consulting. The saccade and fixation targets were projected onto the wall with three red low-power laser lights. The lights were projected onto a matte white wall 1.5–3.5 m away from the participant’s eyes. All saccadometry was conducted under darkened conditions as per a published pilot study by our research group (28). The saccadometer automatically adjusted for minor deviations in distance between the participant’s forehead and the distance to the wall so long as it was within the range defined above. It also automatically marked and excluded intervening blinks.

All participants were guided through calibration and tutorial sessions prior to the antisaccade task. Participants who were myopic or hyperopic were asked to wear contacts to ensure corrected visual acuity for the saccade tests or were examined to ensure they could see all three saccadic targets clearly and without horizontal diplopia before proceeding. The antisaccade task was preprogramed into the saccadometer by the manufacturer. Participants were shown a red center light, after which it jumped either to the left or to the right (fixed probability 50%) with 10° horizontal displacement. There was a fixed foreperiod of 1000 ms and a random foreperiod with a maximum of 1000 ms. They were instructed to look at the center light. When it jumped to the left or to the right, participants were instructed to look in the mirror-opposite location as accurately and quickly as they could. The waiting and trial break time for each saccade was 2000 and 1000 ms, respectively. We performed the gap antisaccade task, in which the center light turned off before presentation of the antisaccade target. Each participant demonstrated understanding of the expected antisaccade task before recording of the data started. Nine acute mTBI participants returned for their second visit, but antisaccade data could not be collected for one participant (*n* = 8).

Raw saccadometry data were transferred to a computer with LatencyMeter Software version 5.2. The software and integrated hardware automatically accounted for eye blinks and head movement during its preprocessing. Saccades with latencies <50 ms (anticipatory saccades) and >600 ms (increased latency due to inattention/possible attempt at malingering) were excluded from the analysis, forming the lower and upper bounds of antisaccade latency, respectively. Saccade trials falling within these bounds were defined as *accepted*. We attempted to make a more homogeneous and reliable subset of trials using this method.

The antisaccade directional accuracy was operationally defined by the number of accepted antisaccades in the correct (anti-) direction for each session. After individual saccadic trials were screened based on the criteria above, intraparticipant median latencies were calculated across trials for each participant at each visit. The interparticipant means of these median values were used for statistical comparisons between groups. An analogous set of dependent variables were calculated for incorrect prosaccade errors, which fit within the defined latency range for all the three groups across all the visits.

### Neuroimage Acquisition and Processing

We used a 2.4 mm × 2.4 mm × 3.0 mm voxel interleaved echo-planar acquisition, on a 3.0-T Siemens Skyra Magnetom (204 coil elements), with 12 non-collinear diffusion directions and one *b*_1_ = 0 reference scan at 3.0 mm slice thickness. The *b*_2_ factor = 1000 s/mm^2^. For acute patients, the majority of the MRI scans were conducted on the second visit (*n* = 9) so the neuroimage correlational analyses were conducted using data from this visit. For control participants, the majority of MRI scans were also conducted on the second visit (*n* = 7) to match the acute patients.

Diffusion MR images were preprocessed and analyzed using the Functional Magnetic Resonance of the Brain (FMRIB) FSL Toolbox (FMRIB Software Library, Release 5.0, 2012, University of Oxford, England on OS X 10.9) (29). Raw NIfTI images were inspected manually for any gross acquisition abnormalities. Scans that developed excessive gross distortions due to severe head motion or dental braces during acquisition, or incomplete scans were excluded from further analyses. Images were eddy-corrected using the EDDY program in the FSL toolbox to reduce distortion effects and skull-stripped using the Brain Extraction Tool (BET) from the FSL toolbox to exclude the non-brain voxels. Using FSLVIEW, each scan was manually checked for eddy correction and brain extraction errors. Binary masks were manually drawn for each scan using FSLVIEW and used for diffusion tensor fitting using DTIFIT. Parameters included the original eddy-corrected diffusion weighted image, the manually drawn binary mask, and the eigenvalue/eigenvector files, respectively.

Four scans could not be included in the DTI analysis. Two PTS participants were excluded due to severe distortion during acquisition of the magnetic resonance image. Two control participants were not included in the analysis because the accompanying eigenvector and eigenvalue files could not be generated from the accompanying DTI scan and because of an adverse event, respectively. We used tract-based spatial statistics (TBSS) to generate the fractional anisotropy and mean diffusivity images necessary for further analysis and calculation of the ROI-based values. We defined white matter tracts from the Johns Hopkins University (JHU) DTI Atlas (30). ROI-based mean FA and MD values were then used in the further statistical comparisons. Mean diffusivity maps were generated in a similar fashion to fractional anisotropy, but the final processing step was completed using a custom TBSS script, which projected the mean diffusivity maps onto the original fractional anisotropy data in order to generate a skeletonized mean diffusivity map. MD ROI values were calculated in a similar fashion using the binarized templates from the JHU Atlas. This process was completed separately for an acute versus control comparison and a PTS versus control comparison.

### Symptom Burden and Executive Function Assessment

Participants completed the RPQ and the symptom assessment scale of the Sport Concussion Assessment Tool Version 3 (SCAT3). Participants also completed the Center for Epidemiological Studies – Depression (CES-D) scale to assess affective state. To measure executive function, a core set of tests within a larger battery was used, including the Stroop color and word test, the trail making test, and the phonemic fluency score from the Montreal Cognitive Assessment (MoCA), English Version 3. The Stroop color and word test was administered with modified instructions from Golden (1945), reviewed by Dr. Grant Killian (31).

### Statistical Analyses

Cross-sectional antisaccade and neuropsychological testing data were analyzed using analysis of variance (ANOVA) with Fisher’s least significant difference (LSD) in *post hoc* comparisons. Longitudinal antisaccade data were analyzed using paired *t*-tests. Neuroimaging data were analyzed using Wilcoxon rank-sum and Welch’s *t*-tests depending on the data distribution. Pearson correlations were used for parametric data and Spearman correlations for non-parametric data. Statistical analyses were conducted in SAS 9.4 (SAS Institute, Inc., Cary, NC, USA) and GNU R 3.0.3 for Windows (R-Core-Team, 2014).

## Results

### Participant Characteristics

Basic demographic characteristics of our participant groups are in Table 1, and more information is in Supplementary Table S1 in Supplementary Material. A significant proportion (64%; *n* = 7) of the acute mTBI group had a history of previous concussion or head injury, and about half had comorbid medical conditions, such as bone, cardiovascular, respiratory, or renal disease. About 64% (*n* = 7) of the acute group needed corrected vision due to myopia or hyperopia. The PTS group had their single visit a median of 8.2 months after their mTBI. A third of the PTS patients endorsed a history of previous concussion or head injury; about half (*n* = 8) had comorbid medical conditions.

**Table 1:** **Study participant demographic characteristics of acute mTBI, PTS, and healthy control groups**.

Participant group	N (first visit)	Mean age (SD)	Sex (M:F)	Education (full years from Grade 1) mean (SD)	GCS score
Acute mTBI V1	11	36.5 (17) years	7:4	15.8 (5) years	All 15 in Emergency Department (ED) All 15 at recruitment
PTS V1	15	42.5 (15) years	5:10	14.9 (4) years	30 min post injury or later upon reassessment in ED (based on ED chart or clinical note describing injury): GCS 13: 1; GCS 14: 1; GCS 15: 13. All 15 at recruitment
Healthy control V1	10	35.5 (21) years	5:5	15.4 (1) years	All 15

### Antisaccade Latency and Error Duration Is Detrimentally Affected after Injury

**Figure 2:**
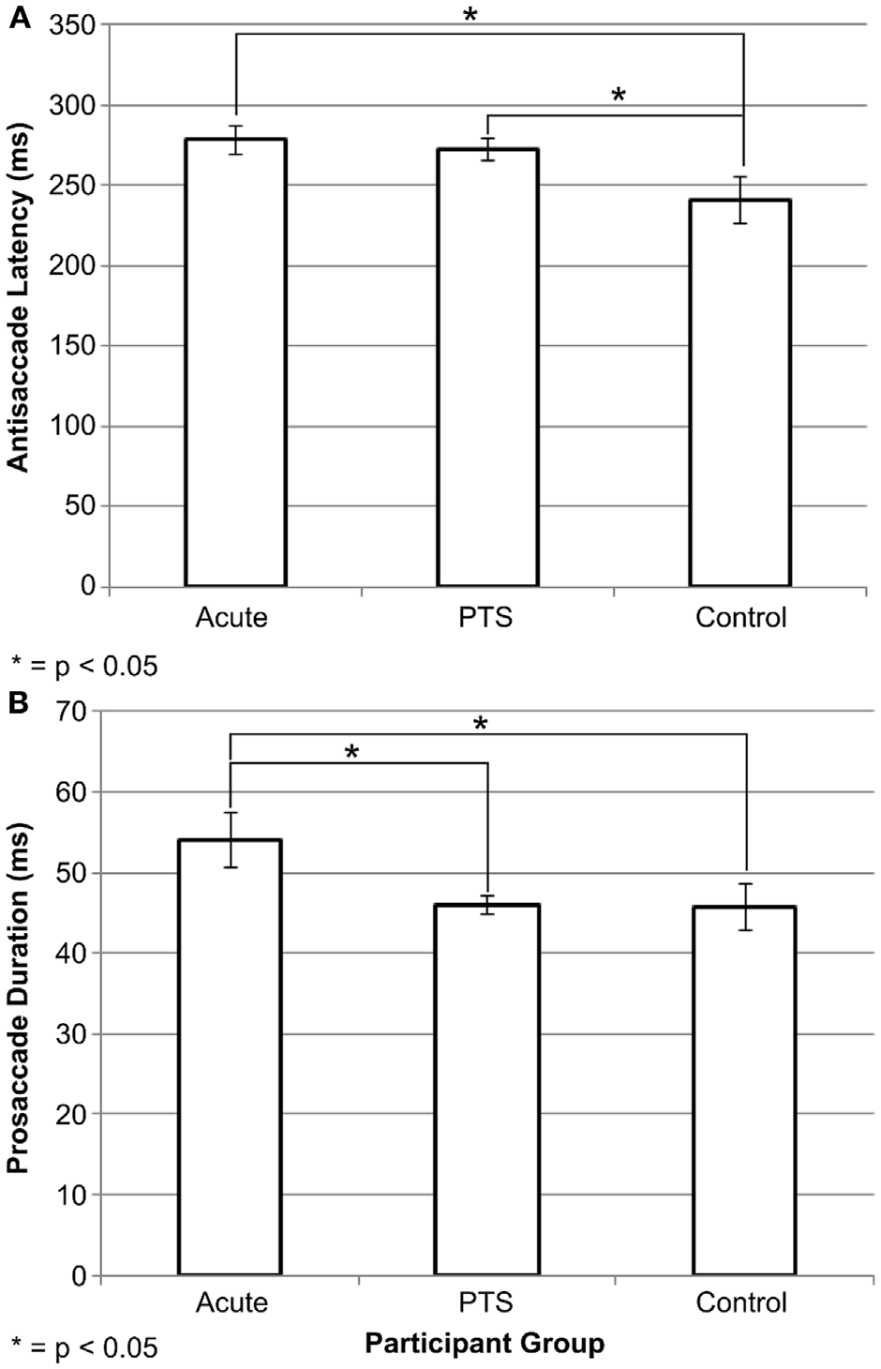
**Antisaccade differences between study groups**. Graphs of **(A)** antisaccade latency performance and **(B)** error duration across participant groups. Acute mTBI and PTS groups had significantly greater antisaccade median latency than the control group. The acute mTBI group had greater error duration than the PTS and control groups. Error bars are ±SEM.

Saccadometry data are in Table 2, and more details are in Supplementary Tables S2 and S3 in Supplementary Material. There was a significant omnibus difference on median antisaccade latency, *F*(2, 31) = 3.65, *p* = 0.04. *Post hoc* LSD comparisons suggested that acute mTBI participants [mean = 278.36 (SD = 28.8)] and PTS participants [272.86 (26.1)] had greater latency than the control group [241.17 (45.2)]. Figure 2A illustrates this result. There was a significant overall difference in mean error duration, *F*(2, 31) = 3.63, *p* = 0.038. *Post hoc* LSD comparisons suggested that acute mTBI participants [54.07 (11.3)] had greater error duration than the PTS group [45.96 (3.99)] and the control group [45.73 (9.10)]. Figure 2B illustrates this finding. Cross-sectional comparisons on the first visit between acute mTBI, PTS, and control groups were not significant for number of accepted antisaccades, antisaccade latency, mean duration, mean amplitude, and mean peak velocity. There were no significant differences within the acute participants between the first and second visits on all antisaccade performance metrics. In healthy controls, the error median latency was significantly greater in the first visit [189.31 (40.18)] compared to the second visit [168.38 (24.59)] [*t*(7) = 3.155, *p* = 0.016]. Other antisaccade performance metrics were not significant between the two visits of control participants.

**Table 2:** **Cross-sectional comparisons of antisaccade performance in the first visit**.

Mean (SD)	Acute mTBI visit 1	PTS visit 1	Healthy control visit 1	Results
*N*	11	14	9	
Antisaccade number accepted	51.9 (25.3)	55 (21.6)	72.2 (26.6)	*F*(2, 31) = 2.01, *p* = 0.15
Antisaccade median latency (ms)	278.36 (28.8)	272.86 (26.1)	241.17 (45.2)	*F*(2, 31) = 3.65, *p* = 0.04
				*Post hoc*: acute > control (confidence limits 7.05, 37.20), PTS > control (−60.34, −3.04)
Antisaccade mean duration (ms)	85.12 (36.0)	68.33 (32.5)	63.45 (17.2)	*F*(2, 31) = 1.46, *p* = 0.25
Antisaccade mean amplitude (°)	15.48 (5.98)	13.04 (6.14)	12.58 (5.36)	*F*(2, 31) = 0.75, *p* = 0.48
Antisaccade mean peak velocity (°/s)	387.46 (154.38)	440.92 (259.76)	437.61 (143.88)	*F*(2, 31) = 0.25, *p* = 0.78
Error median latency (ms)	178.64 (1.36)	182.29 (28.9)	193.17 (39.3)	*F*(2, 31) = 0.69, *p* = 0.51
Error mean duration (ms)	54.07 (11.3)	45.96 (3.99)	45.73 (9.10)	*F*(2, 31) = 3.63, *p* = 0.038
				*Post hoc*: acute > PTS (1.29, 14.9), acute > control (0.73, 15.6)
Error mean amplitude (°)	9.92 (1.47)	9.36 (2.09)	8.41 (2.05)	*F*(2, 31) = 1.58, *p* = 0.2214
Error mean peak velocity (°/s)	432.53 (75.95)	470.90 (249.19)	406.85 (101.53)	*F*(2, 31) = 0.39, *p* = 0.6808

### Diffusion Tensor Imaging Identified Correlations with Antisaccades

**Figure 3:**
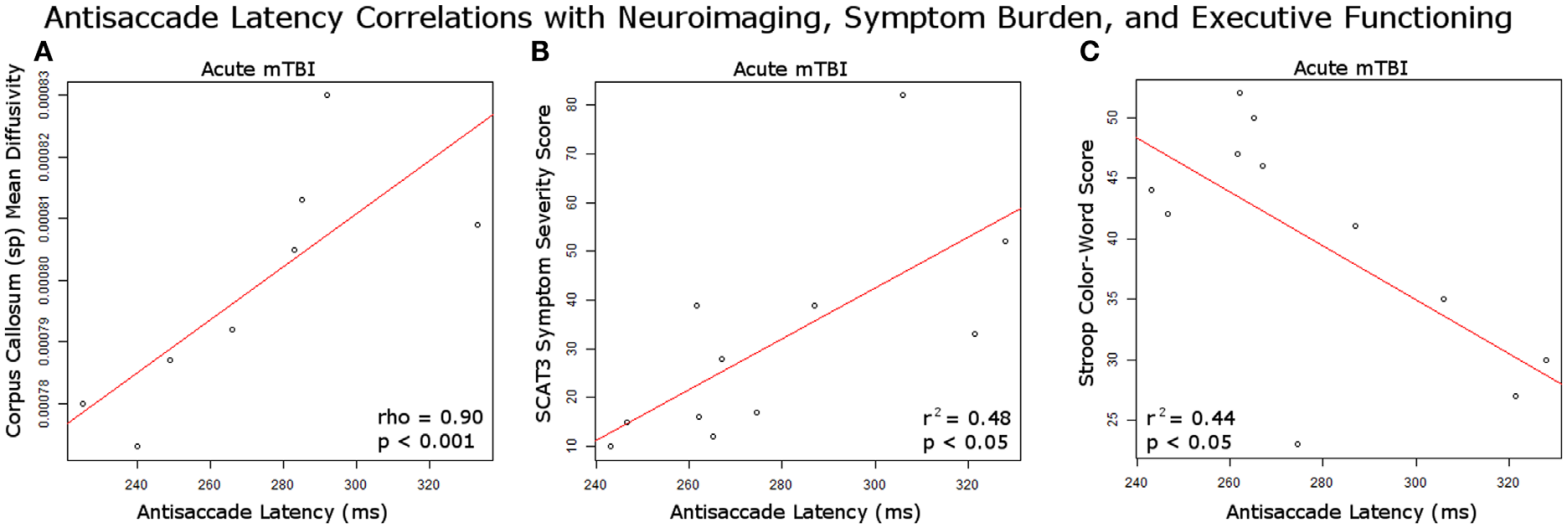
**Antisaccade correlations with study measures**. **(A)** Significant positive correlation at second visit between acutely injured white matter integrity in the corpus callosum splenium and antisaccade latency performance; **(B)** significant positive correlation at first visit between SCAT3 symptom severity score and antisaccade median latency performance in acute mTBI patients; **(C)** significant negative correlation at first visit between Stroop color-word score and antisaccade latency in acute mTBI patients.

A significant positive relationship was identified between the mean diffusivity in the splenium of the corpus callosum and the median antisaccade latency at the second visit for acute mTBI patients [Rho (8) = 0.9048, *p* = 0.00045] (Figure 3A). There was a significantly greater mean diffusivity in the acute (median, Mdn = 0.000805) group compared to the control group (Mdn = 0.000766) in the splenium of the corpus callosum (*p* = 0.03112). There was a significantly lower mean diffusivity in the PTS (*M* = 0.000743) group compared to the control group (*M* = 0.000761) in the corticospinal tract (*p* = 0.04917). There were no other significant differences between groups in fractional anisotropy and in mean diffusivity. There were no significant correlations between right corticospinal tract mean diffusivity and antisaccade median latency/error duration for PTS patients at their only visit.

### Symptom Burden and Executive Functioning Correlated with Antisaccades

There was a significant positive correlation between antisaccade median latency and SCAT3 symptom severity score, *r*^2^ = 0.480, *p* = 0.02 in the acute mTBI patients at their first visit (Figure 3B). There was a significant negative correlation between antisaccade median latency and stroop color-word score, *r*^2^ = 0.439, *p* = 0.03 in the acute mTBI patients at the first visit (Figure 3C). There was a significant difference between groups on the Stroop color-word score, *F*(2, 33) = 6.59, *p* = 0.0039. *Post hoc* comparisons indicated that acute mTBI participants [39.7 (9.7)] scored worse compared to controls [52.1 (12.0)] and PTS participants [39.1 (7.1)] also scored worse than controls.

SCAT3 total score *F*(2, 33) = 30.8, *p* < 0.0001 was significantly greater in the acute mTBI group [13.3 (4.76)] and PTS group [16.3 (5.91)] compared to controls [1.3 (2.26)]. SCAT3 symptom severity score *F*(2, 33) = 13.9, *p* < 0.0001 was also significantly different between all the three groups in ascending order, starting with controls [2.3 (3.95)], then to acute mTBI patients [31.18 (21.6)], and then to PTS patients [52.87 (31.0)]. Analysis of RPQ total score indicated significant differences in symptom reporting cross-sectionally between participant groups, *F*(2, 33) = 40.5, *p* < 0.0001. All the three groups were significantly different from one another in ascending order, starting with controls [1.3 (3.47)], then acute mTBI [18 (10.3)], and finally PTS [40.53 (14.0)]. Stroop color performance was ­significantly impaired between groups *F*(2, 33) = 4.67, *p* = 0.0164. *Post hoc* comparisons indicated that acute mTBI participants [75.8 (17.3)] scored worse compared to controls [89 (13.6)] and PTS participants [72.4 (10.2)] also scored worse than controls.

## Discussion

### Main Finding

The main finding of this study is that there is a distinguishing characteristic of the antisaccade, which is significantly affected after injury, and that antisaccade performance is correlated with white matter integrity, response interference, and symptom burden.

### Antisaccade Latency and Error Duration Is Detrimentally Affected after Injury

The median antisaccade latency at the first visit was significantly greater in the patient groups compared to controls, but we found no significant difference between acute mTBI and PTS groups at the first visit. Our analyses suggested that antisaccade latency may be a good metric for distinguishing injury from non-injury, although the heterogeneity of mTBI and persistence of symptoms remain a problem. To some extent, this supports what has been found in the literature in cross-sectional comparisons between injury and control groups. Heitger et al. identified significant increases in antisaccade latency in acute mTBI groups within 1 week of injury compared to control groups (20), and Williams et al. also identified similar greater antisaccade latency in severely injured TBI patients compared to controls (32). There was also a greater spread in latency scores within the control group compared to the injury groups in our results. This could mean that there is a natural variation or normal range of antisaccade latency in our control sample (similar to a normal range of blood pressure or body weight in humans). There are also numerous other factors like attention and alertness level, which may affect performance on the antisaccade task. This is important to investigate further in future studies. Overall, as the antisaccade task involves response inhibition, increased latency in the antisaccade task may reflect greater cortical processing time needed to generate the correct oculomotor command. This finding was in line with our initial hypotheses.

There was also a significant omnibus group-wise difference in mean duration of their error trials, with the acute mTBI participants exhibiting greater duration than the PTS and control participants. The duration of a saccade is related to its amplitude and velocity. We found that the average amplitude for all three participant groups were larger than the target amplitude of 10° on the correct antisaccade responses, but overall were smaller than the target amplitude on the prosaccade error responses. It was interesting that a deficit was observed in the error mean duration, but not antisaccade ­duration. It was noted by an early reviewer that our results in the patient group do not conform to the typical main sequence relationships between amplitude and velocity. Heitger et al. (25) attributed their observed deficits in PTS patients in prosaccade and antisaccade duration to damage in subcortical processing (25). Their results suggest that prosaccade mean ­duration can potentially be used to distinguish between acute mTBI and PTS.

Control participants took less time to make an error prosaccade toward the stimulus during the second visit compared to the first visit (Supplementary Material). The results suggest that some antisaccade variables might not be stable over several weeks, even in healthy controls. Further studies are needed to identify the factors influencing antisaccade performance. The difference in antisaccade median latency in the pairwise control comparisons was not significant, suggesting stable measurement over time on this specific antisaccade variable. The error mean duration was also similar between both visits in each group. Hence, not all measurements taken acutely are reliable indicators of change in clinical state.

### Diffusion Tensor Imaging Identified Correlations with Antisaccades

In line with our hypotheses, we identified a significant positive correlation between median latency of antisaccades at the acute mTBI second visit and MD in the splenium of the corpus callosum. There appeared to be a connection between the time necessary for inhibitory processes to take place, generating a successful antisaccade, and integrity of this white matter bundle. This correlation did not hold for PTS patients. Some have attributed PTS to psychosocial aspects of the injury, whereas the acute manifestations are a product of neurobiological changes in reaction to the impact (33). The lack of effective correlations in PTS patients may reflect this underlying disconnect between neurological functioning and the structural neuroanatomy. Similar relationships between antisaccade performance and corpus callosum integrity have been documented in patients with fetal alcohol spectrum disorder in studies where the patients completed antisaccade tasks as well as diffusion MRI using voxel-based DTI analysis methods (34). The antisaccade task requires much greater recruitment of cerebral resources compared to a simple prosaccade task. It is possible that diffuse axonal injury of the many connections serviced by the corpus callosum splenium plays a significant role in the execution of an efficient antisaccade.

The difference we found in MD of the acute group’s corpus callosum splenium compared to the control group partially confirmed our hypothesis that there would be decreased white matter integrity postinjury in the corpus callosum. Significant microstructural damage and change in the corpus callosum in TBI patients over time have been identified (35, 36). As discussed, a recent meta-analysis specifically focusing on the corpus callosum found that DTI analysis of this area was a sensitive marker for microstructural damage after mild TBI (5). Our findings suggest that corpus callosum microstructural alterations are present several weeks after injury in acute mTBI patients compared to healthy controls. The finding that MD was significantly elevated compared to controls supports the hypothesis that there might be increased edema and inflammation in the splenium component of this critical white matter bundle.

There were no significant differences in the genu and body of the corpus callosum, which was surprising as previous studies have identified vulnerability of these areas (5). Further analysis stratifying the impact location for acute mTBI and PTS patients (i.e., frontal, temporal, parietal, or occipital) may provide some mechanistic explanations for the negative findings in these areas, and the DTI analysis as a whole. We did not find significant differences elsewhere. However, traumatic brain injury is a complex biophysical process often characterized by rotational injury as well as blunt trauma (37). There are likely variations in the severity and nature of white matter injury in the mTBI patients that were scanned, which could be further characterized by localizing the area of injury and a review of the structural images for distribution of focal white matter hyperintensities and/or infarcts. Contrary to our hypothesis, there were no significant differences in fractional anisotropy between the acute and control groups in all the ROIs studied. However, FA measures a different aspect of diffusion flow and white matter integrity compared to mean diffusivity and further studies are needed to examine why some differences were seen in MD, and why FA was left unaltered postinjury. Longitudinal studies of DTI have found fluctuations in mean diffusivity differences from the days to a year following injury (7). It is understood that FA and MD will rise and fall over time from the acute stages to chronic stages, and our findings may reflect these natural fluctuations in the corticospinal tract. Even in the acute stages, there is little consensus as to whether FA and MD will increase or decrease, as different findings have been identified between studies (38).

### Symptom Burden and Executive Functioning Correlated with Antisaccades

Correlational analyses identified several associations between antisaccade performance and symptom load/executive performance that were meaningful. We found a positive correlation between antisaccade median latency and SCAT3 symptom load, and a negative correlation between antisaccade median latency and Stroop interference performance at the first visit. The first finding made sense in that poorer antisaccade performance was correlated to increased symptom burden on the SCAT3. Increased antisaccade latency is a disorder of saccadic initiation. The time required for the integration and calculation of the antisaccade signal reflects the time needed to process this task involving higher brain structures and interhemispheric connectivity (10), in part mediated by the corpus callosum. Intact white matter architecture and functioning of the higher cerebral centers are necessary for effective saccadic initiation and normal cognitive functioning. Impaired cognitive functioning and specifically an effective way to cope with response interference may push symptom burden higher in acute mTBI and PTS patients. It remains to be determined why this relationship was only identified for the acute mTBI group and not the PTS group, even though the PTS group reported significantly greater symptom burden on the RPQ and the SCAT3 on almost every question and symptom category.

Since there was a significant negative association between antisaccade performance and Stroop color-word score, a potential mechanistic explanation is that antisaccade performance reflected this executive neurocognitive impairment, which in turn was associated with increased symptom burden on the SCAT3. Currently, there is no validated subscoring system for the symptom assessment scale of the SCAT3. Future work could do factor analyses on the SCAT3 symptom scale to identify a stable cognitive performance subscale on the SCAT3 (similar to the RPQ) and use this subscore to correlate with antisaccade performance and executive functioning. We found that task interference inhibition was impaired in acute mTBI patients with the Stroop test. A similar cognitive process is needed in the brain to generate an effective antisaccade: the participant must *inhibit* the easiest rapid eye movement, an *interfering* one moving toward the stimulus (committing a prosaccade error), and cognitively program an antisaccade, instead rapidly moving the eyes to the mirror-opposite location. The interference deficit between the acute mTBI patients and controls suggest the antisaccade task could potentially be a marker for this specific neurocognitive domain and provides a neuropsychological link to the larger implications of this work.

Connections have been found in past research in PTS patients, between antisaccade performance and executive functioning (39). We could not confirm this relationship in our results. This was surprising, given that the level of executive functioning impairment in PTS (measured by the Stroop color-word score) was similar to the acute group. One possible explanation is that in PTS patients, the changes in microstructure leading to sustained impairment in antisaccade performance were different compared to acute patients. This explanation is supported by our finding that the differences in mean diffusivity were found in the corpus ­callosum in the acute patients, but not in the PTS patients; ­likewise, significant MD differences were identified in the right corticospinal tract in the PTS patients, but not the acute patients. Thus, a difference in the neuroanatomy of PTS may be an explanation for the negative findings here. As discussed in the introduction, the most drastic effects of the neurometabolic cascade happen acutely after injury, but they can ease into permanent damage in the chronic phases. Perhaps, these changes are reflected in our neuroimaging results, but the study’s small sample size limits our conclusions.

In terms of executive functioning, in line with our hypotheses, there was a significant difference between the injured and control groups on the Stroop color-word score, with injured participants covering fewer items in the same amount of time as controls (hence scoring more poorly). The results suggested that the injured groups had significantly impaired interference control as tested by the Stroop interference trial compared to healthy control groups. We did not see significant differences between the acute and PTS group, suggesting similar levels of response inhibition impairment. We found significant differences in the Stroop color score with poorer performance in the injury groups; there have been reports of impaired color score differences in patients with postconcussive syndrome compared to controls (40).

There were no significant differences in phonemic word fluency on the MoCA, and also no significant differences on the Trail Making Test *B* score although TMT-A performance was altered. The finding that differences in TMT-A were observed suggested there are differences in psychomotor speed between groups. White matter structural damage may be the common denominator of deficits in psychomotor speed and the deficits observed in antisaccade latency performance and response inhibition, but more work is required.

Our work supports that antisaccade latency is related to impaired inhibitory functioning, diffuse axonal injury in the corpus callosum, and symptom load. We confirmed significant characteristics of antisaccade functioning, which were affected in injury patients. We also identified deficits in specific types of executive functioning, and identified relationships between antisaccade performance and symptom load. Our findings indicate that it may be possible to distinguish between mTBI participant groups and controls using antisaccades, both in the acute and chronic phases. However, any antisaccade metric that will be used for clinical purposes needs to be selected carefully and must be robust.

### Limitations and Future Directions

We did not include a task to account for malingering on the neuropsychological testing and inflated symptom reporting in the acutely or chronically injured patients. We did not account for depression score and other factors that may affect the relationships studied (age and time since injury, for example). Furthermore, we did not exclude patients who had a comorbid history of posttraumatic stress disorder (PTSD), which affects the psychological aspect of neurocognitive functioning and symptom burden. We also did not account for history of previous concussion due to low sample sizes. We used the latency range of 50–600 ms for our initial screening and identification of “accepted” saccades based on previous work on the step saccade task (28). However, close to half of the antisaccade trial responses ended up falling out of these boundaries, limiting the validity of our conclusions. A new upper and lower bound for antisaccade latencies used for this task may be needed. We averaged the FA and MD values over the whole white matter region of interest (ROI). Thus, it is possible that any individual differences at specific points along the tracts were masked. This limitation was touched upon by Niogi et al. in their review (4) noting the large number of participants needed to adequately power a ROI-based inquiry of DTI changes after mTBI. As reviewers noted, since computerized tomography results are more likely to be used for mTBI, future work may consider any correlations between tomography findings and PTS. Furthermore, this population is inherently heterogeneous and future work may need to account for this variability, and a sensitivity/specificity analysis would be very informative with a larger sample size. Finally, although our experimental design and timing of visits was adjusted to overall clinical course for recovery in mTBI patients and the expected course of recovery in saccadic eye movements, it is possible that this timeframe was not long enough for significant resolution of symptoms in the acute mTBI patients. We also did not correct for multiple comparisons due to the exploratory nature and small sample size of this study. Nevertheless, we think this work is an important step toward identifying the relationship of the antisaccade to other assessment methods used for these patients.

## Conclusion

In conclusion, there were differences in antisaccade median latency and error mean duration between mTBI patient groups and controls. These measures were associated with loss of white matter integrity in the splenium of the corpus callosum; increased antisaccade median latency was also associated with poor performance on executive functioning tasks and greater symptom load. This study adds to the existing literature suggesting that the antisaccade task may have potential to be useful as a neurological marker for acute mTBI, but with our low sample sizes and the exploratory nature of this study further investigation is required.

## Author Contributions

Conception of the work: MC, TS, JT-V, and WT; collection of data: WT and MC; data analysis: WT; data interpretation: MC, TS, JT-V, and WT; wrote initial draft of manuscript: WT; and revisions to the manuscript for significant intellectual content: MC, TS, JT-V, and WT.

## Conflict of Interest Statement

The authors declare that the research was conducted in the absence of any commercial or financial relationships that could be construed as a potential conflict of interest.
