# Supplementary material for: Antisaccadic Eye Movements Are Correlated with Corpus Callosum White Matter Mean Diffusivity, Stroop Performance, and Symptom Burden in Mild Traumatic Brain Injury and Concussion
Source: Front Neurol. 2016 Jan 18;6:271. doi: 10.3389/fneur.2015.00271 (PMC4716139; doi:10.3389/fneur.2015.00271)
Supplement: Supplementary file 1 [file datasheet_1.docx]

Supplementary Material

Antisaccadic eye movements are correlated with corpus callosum white matter mean diffusivity, stroop performance and symptom burden in mild traumatic brain injury and concussion

Windsor Kwan-Chun Ting, Tom A. Schweizer, Jane Topolovec-Vranic, Michael D. Cusimano*

*** Correspondence:** Michael D. Cusimano: injuryprevention@smh.ca

# Supplementary Methods

MTBI is an acute brain injury resulting from mechanical energy to the head from external physical forces. Operational criteria for clinical identification include: (i) 1 or more of the following: Any alteration in mental state at the time of the injury (confusion, disorientation, dazed, slowed thinking, etc.), loss of consciousness for 30 minutes or less, post-traumatic amnesia for less than 24 hours or for events immediately before (retrograde amnesia), and/or other transient/persistent neurological abnormalities such as weakness, loss of balance, change in vision, dyspraxia paresis/plegia [paralysis], sensory loss, aphasia, etc; other focal signs, seizure, and intracranial lesion not requiring surgery; (ii) Glasgow Coma Scale score of 13–15 after 30 minutes post-injury or later upon presentation for healthcare. These manifestations of MTBI must not be due to drugs, alcohol, medications, caused by other injuries or treatment for other injuries (e.g. systemic injuries, facial injuries or intubation), caused by other problems (e.g. psychological trauma, language barrier or coexisting medical conditions) or caused by penetrating craniocerebral injury. (Underlined qualifications from American College of Rehabilitation Medicine) ([Carroll et al., 2004](#_ENREF_1);[Ruff et al., 2009](#_ENREF_2))

# Supplementary Figures and Tables

Supplementary Table 1: Detailed Participant Characteristics

| **Participant Group** | **Acute mTBI** | **PTS** | **Healthy Control** |
| --- | --- | --- | --- |
| **N** | Visit 1: 11  Visit 2: 9 | Visit 1: 15 | Visit 1: 10  Visit 2: 8 |
| **Testing Latency (Mean Days between injury and testing)** | Visit 1: 4.73  Visit 2: 22.56 | Visit 1: 460.2. Median 8.2 months | NA |
| **MRI Conducted on First or Second Visit** | Visit 1: 2  Visit 2: 9 | Visit 1: 15 | Visit 1: 3  Visit 2: 7 |
| **History of Previous Concussion** | Yes: 7  No: 4 | Yes: 5  No: 9  Not sure: 1 | Yes: 0  No: 10 |
| **Dominant Hand** | Left: 0  Right: 11 | Left: 2  Right: 12  Ambidextrous: 1 | Left: 2  Right: 8 |
| **First Language** | English: 9  Farsi: 1  French: 1 | English: 9  Spanish: 2  Russian: 1  Greek: 1  Italian: 1  Arabic: 1 | English: 3  Spanish: 2  German: 1  Japanese: 1  Chinese: 1  Tamil: 1  Ukrainian: 1 |
| **History of Past Eye Injury** | Yes: 0  No: 11 | Yes: 1  No: 14 | Yes: 0  No: 10 |
| **Presence of Other Medical Conditions (comorbidities)** | Yes: 5  No: 6 | Yes: 8  No: 7 | Yes: 3  No: 7 |
| **Alcohol Consumption History (Current or past consumption)** | Yes: 10  No: 1 | Yes: 12  No: 3 | Yes: 6  No: 4 |
| **History of Drinking Problems and Treatment** | Yes: 0  No: 11 | Yes: 0  No: 15 | Yes: 0  No: 10 |
| **Affective Disorder History (anxiety or depression)** | Yes: 2  No: 9 | Yes: 11  No: 4 | Yes: 0  No: 10 |
| **Mean CES-D Score** | M: 13.09  SD : 7.92 | M: 23.2  SD: 10.8 | M: 6.90  SD: 4.46 |
| **Vision Correction History** | Yes: 7  No: 4 | Yes: 14  No: 1 | Yes: 5  No: 5 |

Supplementary Table 2: Results in Longitudinal Comparisons of Antisaccade Performance in Acute mTBI

| **Mean (SD)** | **Acute mTBI**  **V1** | **Acute mTBI**  **V2** | **Results** |
| --- | --- | --- | --- |
| **Antisaccade Number Accepted** | 51.9 (25.3) | 54.5 (27.2) | t(7) = -1.06, p = 0.3253. |
| **Antisaccade Median Latency (ms)** | 286.5 (29.78) | 271.63 (34.24) | t(7) = 2.2064, p = 0.06314. |
| **Antisaccade Mean Duration (ms)** | 76.05 (36.86) | 74.98 (25.17) | t(7) = 0.1042, p = 0.9199. |
| **Antisaccade Mean Amplitude (deg)** | 14.63 (6.20) | 12.45 (2.69) | t(7) = 1.1182, p = 0.3004. |
| **Antisaccade Mean Peak Velocity**  **(deg/s)** | 444.84 (100.08) | 388.63 (81.65) | t(7) = 1.2431, p = 0.2538. |
| **Error Median Latency (ms)** | 177.25 (15.62) | 184.62 (22.81) | t(7) = -0.9304, p = 0.3831. |
| **Error Mean Duration (ms)** | 52.03 (11.2) | 50.68 (7.59) | t(7) = 0.4653, p = 0.6558. |
| **Error Mean Amplitude (deg)** | 10.44 (1.24) | 10.04 (3.46) | t(7) = 0.2664, p = 0.7976. |
| **Error Mean Peak Velocity (deg/s)** | 467.92 (49.15) | 434.12 (128.86) | t(7) = 0.6576, p = 0.5318. |

Supplementary Table 3: Reliability of Antisaccade Performance in Healthy Control Participants

| **Mean (SD)** | **Control**  **V1** | **Control**  **V2** | **Results** |
| --- | --- | --- | --- |
| **Antisaccade Number Accepted** | 72.22 (26.57) | 68.63 (19.95) | t(7) = 1.86, p = 0.1047 |
| **Antisaccade Median Latency (ms)** | 231.44 (36.8) | 218.81 (28.8) | t(7) = 2.104, p = 0.07344. |
| **Antisaccade Latency Rate**  **(ms^-1^)** | 0.00442 (0.000704) | 0.00464 (0.000608) | t(7) = -1.868, p = 0.1039. |
| **Antisaccade Mean Duration (ms)** | 61.54 (17.37) | 65.53 (15.24) | t(7) = -0.5362, p = 0.6084. |
| **Antisaccade Mean Amplitude (deg)** | 11.42 (4.36) | 10.48 (3.07) | t(7) = 0.9097, p = 0.3932. |
| **Antisaccade Mean Peak Velocity (deg/s)** | 417.65 (139.86) | 363.51 (90.51) | t(7) = 1.2356, p = 0.2565. |
| **Error Median Latency (ms)** | 189.31 (40.18) | 168.38 (24.59) | t(7) = 3.155, p = 0.01604. |
| **Error Mean Duration (ms)** | 45.77 (9.73) | 50.35 (7.26) | t(7) = -1.3971, p = 0.2051. |
| **Error Mean Amplitude (deg)** | 8.06 (1.87) | 8.39 (1.31) | t(7) = -0.8069, p = 0.4463. |
| **Error Mean Peak Velocity (deg/s)** | 387.93 (90.0) | 368.58 (51.97) | t(7) = 0.4867, p = 0.6414. |

# Supplementary References

Carroll, L.J., Cassidy, J.D., Holm, L., Kraus, J., Coronado, V.G., and Injury, W.H.O.C.C.T.F.O.M.T.B. (2004). Methodological issues and research recommendations for mild traumatic brain injury: the WHO Collaborating Centre Task Force on Mild Traumatic Brain Injury. *J Rehabil Med***,** 113-125.

Ruff, R.M., Iverson, G.L., Barth, J.T., Bush, S.S., Broshek, D.K., Policy, N.a.N., and Planning, C. (2009). Recommendations for diagnosing a mild traumatic brain injury: a National Academy of Neuropsychology education paper. *Arch Clin Neuropsychol* 24**,** 3-10.
